# Nanomechanical characterization of chemical interaction between gold nanoparticles and chemical functional groups

**DOI:** 10.1186/1556-276X-7-608

**Published:** 2012-10-31

**Authors:** Gyudo Lee, Hyungbeen Lee, Kihwan Nam, Jae-Hee Han, Jaemoon Yang, Sang Woo Lee, Dae Sung Yoon, Kilho Eom, Taeyun Kwon

**Affiliations:** 1 Institute for Molecular Sciences, Seoul, 120-749, Republic of Korea; 2 Department of Biomedical Engineering, Yonsei University, Wonju, 220-710, Republic of Korea; 3 Department of Energy IT, Gachon University, Seongnam, Gyeonggi-do, 461-701, Republic of Korea; 4 Department of Radiology, College of Medicine, Yonsei University, Seoul, 120-749, Republic of Korea

**Keywords:** Au nanoparticle, Dopamine, Surface chemistry, Atomic force microscopy, Lateral force microscopy

## Abstract

We report on how to quantify the binding affinity between a nanoparticle and chemical functional group using various experimental methods such as cantilever assay, PeakForce quantitative nanomechanical property mapping, and lateral force microscopy. For the immobilization of Au nanoparticles (AuNPs) onto a microscale silicon substrate, we have considered two different chemical functional molecules of amine and catecholamine (here, dopamine was used). It is found that catecholamine-modified surface is more effective for the functionalization of AuNPs onto the surface than the amine-modified surface, which has been shown from our various experiments. The dimensionless parameter (i.e., ratio of binding affinity) introduced in this work from such experiments is useful in quantitatively depicting such binding affinity, indicating that the binding affinity and stability between AuNPs and catecholamine is approximately 1.5 times stronger than that between amine and AuNPs. Our study sheds light on the experiment-based quantitative characterization of the binding affinity between nanomaterial and chemical groups, which will eventually provide an insight into how to effectively design the functional material using chemical groups.

## Background

Surface chemistry has played a critical role in designing functional nanomaterials for their biological or medical applications such as drug delivery, molecular therapeutics, and diagnostics [1,2]. In particular, the surface modification of a nanoparticle is of great importance to enhancing functionality in terms of target affinity [3-5], imaging contrast [3,4,6,7], and curative power [8]. For instance, magnetic nanoparticles chemically modified with chemical functional groups or moieties (e.g., ligand and receptor) have been utilized for high-resolution MRI, which is useful in cancer diagnostics since the chemical modification using chemical functional groups or moieties leads to improved targetability and imaging contrasts [3,6,7]. Moreover, gold nanoparticles (AuNPs) functionalized with chemical functional groups or moieties have been recently used to enhance photocatalytic performance [9], to form 3D networks of functionalized AuNPs [10], and to sensitively detect specific biological molecules (e.g., DNA) [11-13] and cancerous single cells [14,15].

Dopamine hydrochloride (DOPA) has recently been considered as a chemical linker that allows for efficient surface chemistry useful in not only inorganic materials (e.g., nanoparticles) but also biological materials (e.g., tissue) due to its excellent adhesive property and biocompatibility [16,17]. In particular, DOPA has been reported as a chemical linker that is useful not only in the chemical modification of the surfaces of nanomaterials such as nanoparticles [18,19], graphene oxide sheet [20], and carbon nanotubes [21], but also in improving binding affinities such as protein-peptide cross-linking [22], cellular adhesion to substrate [23], osteoconduction [24], and hemostatic adhesive in segmentectomy [25]. Despite the broad application of DOPA to surface chemistry using mutual interaction between DOPA and nanomaterials (e.g., nanoparticle), such an interaction has been poorly understood and not yet studied thoroughly. Since the surface modification of nanomaterials using DOPA typically employs a noncovalent conjugation (e.g., coordinate bonding, hydrophobic and electrostatic interactions, etc.) [6,26], it is essential to establish an experimental framework that allows for measuring a weak binding affinity corresponding to such a noncovalent conjugation, which is useful in the development of drug carrier due to the fact that noncovalent conjugation enables the excretion of waster matter from the human body after the drug carrier completes the function of drug delivery or bioimaging [6,7,27,28].

In this work, we have quantitatively studied a chemical interaction between nanoparticles and chemical functional groups (e.g., DOPA and amine functional group) using experimental toolkits such as cantilever bioassay [29-32], PeakForce Quantitative Nanomechanical Property Mapping (PeakForce QNM) [33,34], as well as lateral force microscopy (LFM) [35-38]. In a recent decade, cantilever bioassays have been widely utilized for quantitative understanding of molecular interactions on the surface by measuring the bending deflection change [39,40] and/or shifts in resonance [29,41]. Moreover, a cantilever has been also employed to measure physical quantities such as temperature [42], quantum state [43], and surface stress [29,44]. We have shown that a cantilever whose surface is functionalized with specific chemical functional groups (DOPA or amine functional group) allows us to quantitatively characterize the binding affinity between nanoparticles and such chemical functional groups. Furthermore, LFM has recently been taken into account for deciphering the molecular interactions by estimating a frictional force that occurs due to breakage of such molecular interactions [35,38]. In our study, we have employed LFM enabling the movement of a nanoparticle, which is chemically interacting with chemical functional groups on the surface, in order to quantitatively understand the binding affinity between nanoparticle and chemical functional groups by measuring the frictional forces required to break the binding between the nanoparticle and chemical functional groups. In addition, we have also measured the adhesion force between nanoparticles and chemical functional groups using atomic force microscopy (AFM), particularly the PeakForce QNM module. We have shown that the noncovalent interaction between nanoparticles and specific chemical functional groups can be quantitatively studied using the aforementioned experimental techniques (i.e., cantilever assay, PeakForce QNM, and LFM) and that catecholamine (i.e., DOPA) is a chemical functional group useful in the surface modification of nanomaterials (e.g., nanoparticle) due to its excellent binding affinity.

## Methods

### Materials and sample preparation

All materials including gold nanoparticle (G1652, approximately 20 nm in size) and dopamine hydrochloride ((HO)_2_C_6_H_3_CH_2_NH_2_·HCl) were purchased from Sigma-Aldrich (St. Louis, MO, USA). A silicon (Si) microcantilever (TESP, Bruker, Madison, WI, USA) was first rinsed by piranha solution (50% of sulfuric acid and 50% of hydrogen peroxide). The cantilever was immersed for 25 min into a 3-aminopropyltrimethoxysilane (APTMS) solution (200 μl/ethanol of 5 ml) for amine functionalization and then carefully washed by ethanol and pure water. The aminated surface of the cantilever (*S*_A_) was immersed into the AuNP suspension (approximately 0.01% as HAuCl_4_) for 30 min for the preparation of AuNP-*S*_A_ (i.e., AuNP attached to amine-modified surface). In the case of DOPA-functionalized surface (*S*_D_), the aminated microcantilever was treated with glutaraldehyde (GA, 10% in phosphate-buffered saline (PBS)) for 30 min for surface activation and then immersed into the DOPA solution (65 mM in PBS at pH 7.4) for 10 h [16]. Consequently, the DOPA-functionalized cantilever was immersed into the AuNP-dissolved solution for the preparation of AuNP-*S*_D_ (i.e., AuNP bound to DOPA-functionalized surface). All experiment was conducted at room temperature.

### Analysis of surface chemistry

Scanning electron microscopy (SEM) imaging was obtained using JSM-6500 F (JEOL, Tokyo, Japan). The number of AuNPs in the SEM images was accurately counted by ImageJ software (NIH, Bethesda, MD, USA). X-ray photoelectron spectroscopy (XPS) analysis was implemented with Escalab 220i-XL (Thermo VG, Hastings, UK). The sampling area was 5 mm × 5 mm in a vacuum of 1.0 × 10^−9^ mbar with calibration of C 1 *s* (285 eV). To measure the resonant frequency shift of the cantilever due to AuNP binding onto the cantilever surface, the samples were dried overnight in each fabrication process. The resonant frequency of the cantilever is measured using the Nanoscope V controller (Veeco, Santa Barbara, CA, USA).

### Measurement of adhesion/friction forces

PeakForce QNM was used to measure the adhesion between AuNPs and chemically functionalized surface using the BioScope Catalyst (Veeco). For PeakForce QNM imaging, we have used a cantilever, particularly ScanAsyst Air probes (*k*_N_ = 0.58 N/m; Bruker) in 22.2°C and 38% humidity. For LFM imaging, we have employed various AFM cantilever tips (i.e., SNL-10, ScanAsyst Air, ScanAsyst Fluid, Bruker) with their stiffness in the range of 0.1 to 1 N/m. LFM images were obtained by scanning the sample in contact mode with a scan size of 2 × 2 μm^2^, scan rate of 0.5 Hz, and a set point of 1 V. The detached AuNPs from the surface was confirmed by using PeakForce QNM imaging. All AFM, LFM, and PeakForce QNM images were analyzed with NanoScope Analysis software (Bruker).

**Figure 1:**
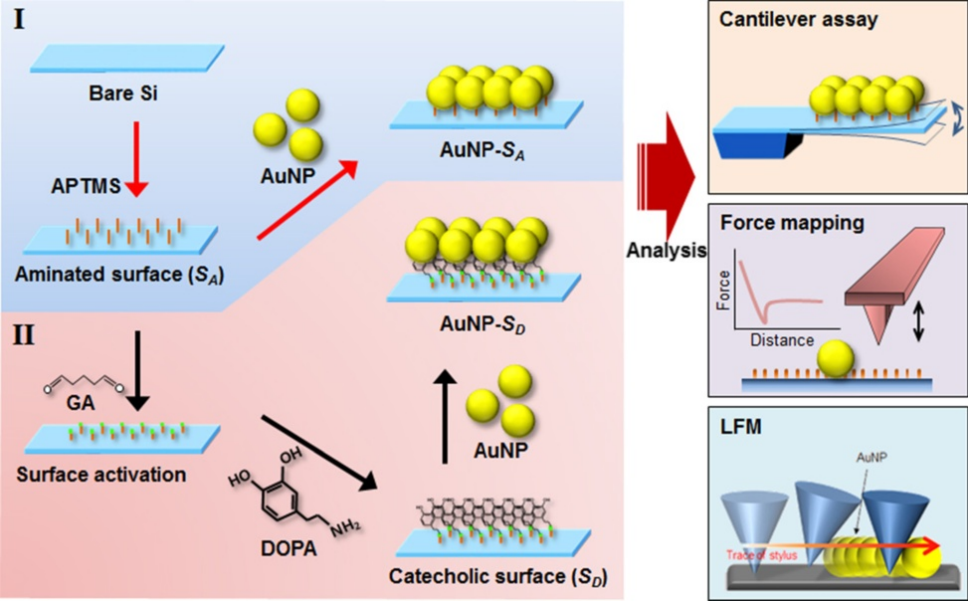
**AuNPs attached onto silicon substrate using chemical functional group.** Schematic illustration of the AuNP-coating procedure on the Si substrate and characterization regimes for the chemical interaction between AuNPs and chemical functional groups. (**I**) AuNPs immobilized on aminated surface (AuNP-*S*_A_); (**II**) AuNPs immobilized on catecholic surface (AuNP-*S*_D_) functionalized with dopamine (DOPA). Each sample is quantitatively characterized by experimental toolkits such as cantilever bioassay, PeakForce QNM, as well as lateral force microscopy (LFM).

We have prepared the silicon surface onto which the AuNPs were attached using chemical functional group (i.e., amine or DOPA), as shown in Figure 1, in order to study the chemical interaction between the nanoparticle and chemical functional group. In particular, we have studied such chemical interaction using various experimental tools such as cantilever assay, LFM, and PeakForce QNM as described above.

## Results and discussion

### Characterization of the AuNPs and surface chemistry of *S*_A_ and *S*_D_

**Figure 2:**
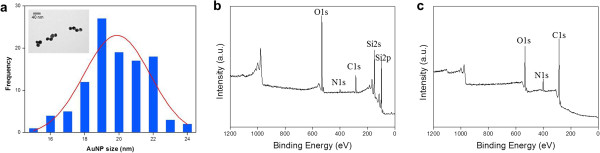
**Characteristics of the AuNPs and surface chemistry of*S***_**A**_**and*S***_**D**_**.** Histogram of AuNP diameters (**a**) and XPS survey spectra of (**b**) the aminated Si substrate (*S*_A_) and (**c**) DOPA-functionalized Si substrate (*S*_D_). Inset in (a): TEM images of the AuNPs.

The size distribution of AuNPs that were used in our experiment was obtained based on transmission electron microscopy (TEM) images as shown in Figure 2a. In particular, the mean diameter of the AuNPs is given as 19.4 nm, and the standard deviation is estimated as 2.2 nm. Moreover, we have confirmed the surface chemistry of the substrate, i.e., chemical functional groups formed on a silicon surface, by using XPS analysis. Figure 2b,c shows the XPS survey of the formation of -NH_2_-Si (panel I in Figure 1) and DOPA-Si (panel II in Figure 1). The N 1 *s* signal appeared in the survey spectrum as the -NH_2_ monolayer was being fabricated on the Si substrate. In the following step, the DOPA was chemically adsorbed onto the amine-functionalized surface and polymerized to form the dopamine layer [45]. The XPS survey shows N 1 *s* and C 1 *s* peaks (Figure 2c) that are higher than those of the -NH_2_-Si sample (Figure 2b). Also, Si 2 *s* and Si 2*p* peaks disappeared in the DOPA-Si sample because of the thickness of the DOPA film, implying the well-formed DOPA layer with self-assembled monolayer [45-47]. 

### Indirect measurement of the binding affinity between AuNPs and chemical functional groups

**Figure 3:**
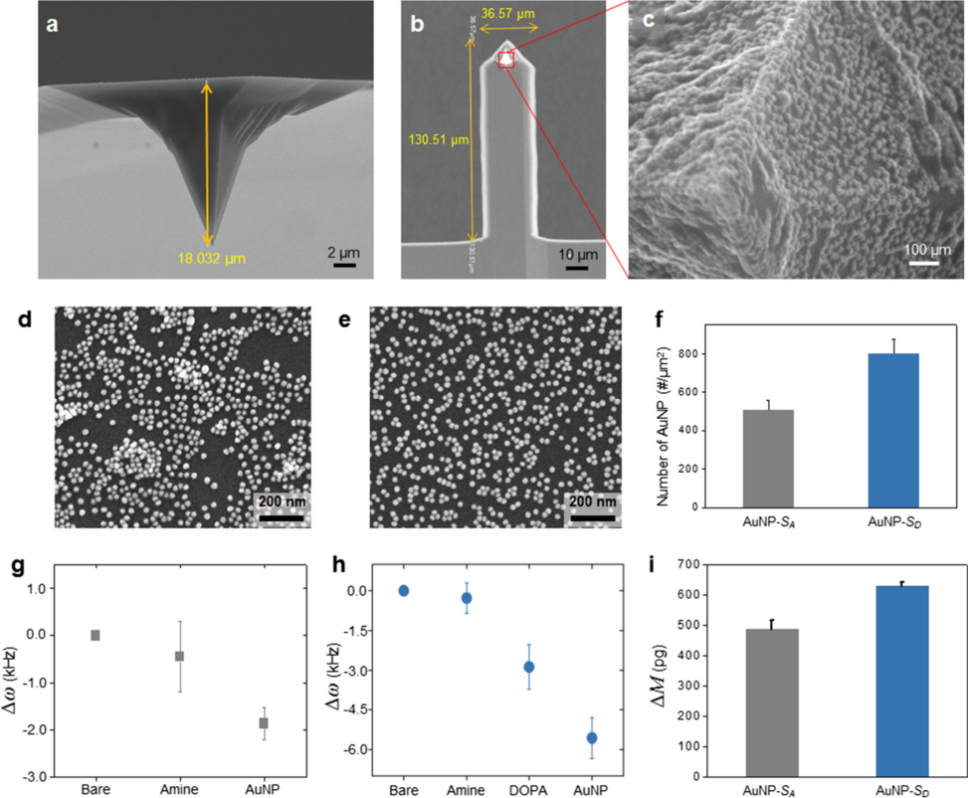
**Characterization of the surface chemistry of the samples such as AuNP-*S***_**A**_**and AuNP-*S***_**D**_**.** (**a, b**) SEM images of the microcantilever functionalized with DOPA molecules used in the cantilever bioassay. The dimensions of the microcantilever such as stylus height, cantilever length, and width are shown. (**c**) The magnified image of the stylus vertex shows uniformly coated AuNPs on the entire microcantilever. (**d, e**) SEM images of the AuNPs immobilized on the Si substrate functionalized with amino groups and DOPA molecules, respectively. (**f**) Plot of the average number of AuNPs from the SEM images (*n* = 5). (**g, h**) Resonant frequency shift of the microcantilever in every step of the fabrication procedure is measured in air (see Table 1). (**i**) Plot of the total mass of AuNPs bound to the chemically modified surface measured from the frequency shift of a cantilever due to AuNP binding (*n* = 3).

We have investigated the binding affinity between AuNPs and the silicon surface chemically modified with a functional group (i.e., amine or DOPA) by measuring the number of AuNPs attached on the chemically modified silicon surface. Here, the DOPA-modified surface is denoted as *S*_D_, whereas we denote the amine-functionalized surface as *S*_A_. It should be also noted that the AuNPs attached to *S*_D_ exhibit uniform shape and size, as shown in Figure 3a,b,c. It is interestingly shown that the AuNPs attached to *S*_A_ were locally aggregated, while the AuNPs immobilized on *S*_D_ were distributed with relatively high uniformity (Figure 3d,e), which suggests that the AuNPs were functionalized as a uniform monolayer onto *S*_D_. It is attributed to the fact that the local aggregation of AuNPs onto *S*_A_ is highly related to the molecular structure of APTMS, which leads to the formation of amine functional group as a disordered layer on the surface and, consequently, the decrease in the uniformity of surface functionalization and large variation of surface density of AuNPs [48]. On the other hand, a uniform attachment of AuNPs onto *S*_D_ is attributed to GA acting as a linker molecule between the surface and DOPA such that the linker molecule allows for an ordered formation of DOPA on a silicon surface. The uniform distribution of the attached AuNPs on the surface is due to the electrostatic repulsion between nanoparticles. Meanwhile, the uniformity of the functionalized molecules is a critical factor in determining the binding affinity of AuNPs [48] because the uniform distribution of functional molecules is *a priori* requisite to optimize the binding affinity between the surface and AuNPs. Although there are small aggregates of AuNPs locally even in the AuNP-*S*_D_ samples, the binding affinity between the surface and AuNPs is clearly shown in the electron microscope imaging assay. The number of AuNPs attached onto either *S*_A_ or *S*_D_ (denoted as *N*_A_ or *N*_D_, respectively) can be used as a quantity that represents the binding affinity. Based on the SEM images of AuNPs attached to either *S*_A_ or *S*_D_, it is found that *N*_A_ = 503 ± 54 (mean ± standard deviation) per unit area of 1 μm^2^, whereas *N*_D_ = 798 ± 75 per unit area of 1 μm^2^ (Figure 3f). This clearly elucidates that *S*_D_ exhibits higher binding affinity to AuNPs than *S*_A_. For quantitative comparison, we have introduced a dimensionless measure defined as *R*_N_ *= N*_D_/*N*_A_. This *R*_N_ ratio can be used as a dimensionless quantity useful in representing the binding affinity (for more detail, see below). 

Now, we have studied binding affinity using cantilever assay that allows for measuring the total mass of AuNPs attached to the surface of the microcantilever. For such a study, we have prepared cantilevers whose surfaces are chemically modified by an amine group or DOPA, respectively. It is shown in Figure 3g,h that the surface modification of cantilevers using amine group or DOPA reduces their resonant frequencies, which is attributed to the weight of the functionalized chemical groups (i.e., amine or DOPA). We have observed that the binding of AuNPs onto the amine- or DOPA-immobilized surface of the cantilever significantly decreases the resonant frequency of such a cantilever (Table 1). The total weight of AuNPs chemically bound to the cantilever can be estimated from the measured frequency shift due to AuNP binding to the cantilever. In particular, the relationship between the total mass of AuNPs bound to the cantilever and the frequency shift is represented in the form Δ*ω*/*ω*_0_ = (1/2)(Δ*M*/*M*_c_), where Δ*ω* is the resonant frequency shift due to AuNP binding onto the cantilever, Δ*M* is the total mass of AuNPs chemically attached to the cantilever, and *ω*_0_ and *M*_c_ represent the resonant frequency and mass, respectively, of the microcantilever whose surface is chemically modified. It is found that the total mass of AuNPs attached to the amine-modified cantilever surface is estimated as Δ*M*_A_ = 488 ± 10 pg, while the total mass of AuNPs bound to the DOPA-functionalized cantilever surface is measured as Δ*M*_D_ = 630 ± 27 pg (Figure 3i). This clearly demonstrates that the DOPA-modified surface exhibits stronger binding affinity to AuNPs than the amine-modified surface. As in the previous paragraph, we have introduced the dimensionless quantity *R*_M_ defined as *R*_M_ = Δ*M*_D_/Δ*M*_A_, which allows for quantitative comparison. It is interestingly shown that *R*_M_ is very close to *R*_N_, as anticipated (i.e., *R*_M_ = approximately 1.3 and *R*_N_ = approximately 1.6). This confirms that the dimensionless quantities *R*_M_ and *R*_N_ are useful parameters that allow for quantifying the binding affinity between the chemically modified surface and AuNPs.

**Table 1:** The resonant frequency shift of AFM cantilevers in cantilever assay

	**Bare**	***S***_**A**_	***S***_**D**_	**AuNP coating**
AuNP-*S*_A_ (kHz)	300.2 ± 32.3	299.8 ± 31.7		298.4 ± 32.6
AuNP-*S*_D_ (kHz)	279.8 ± 3.5	279.5 ± 3.8	276.9 ± 4.0	274.2 ± 4.0

Standard deviations are derived from three independent measurements.

### Direct measurement of the binding affinity between AuNPs and chemical functional groups

**Figure 4:**
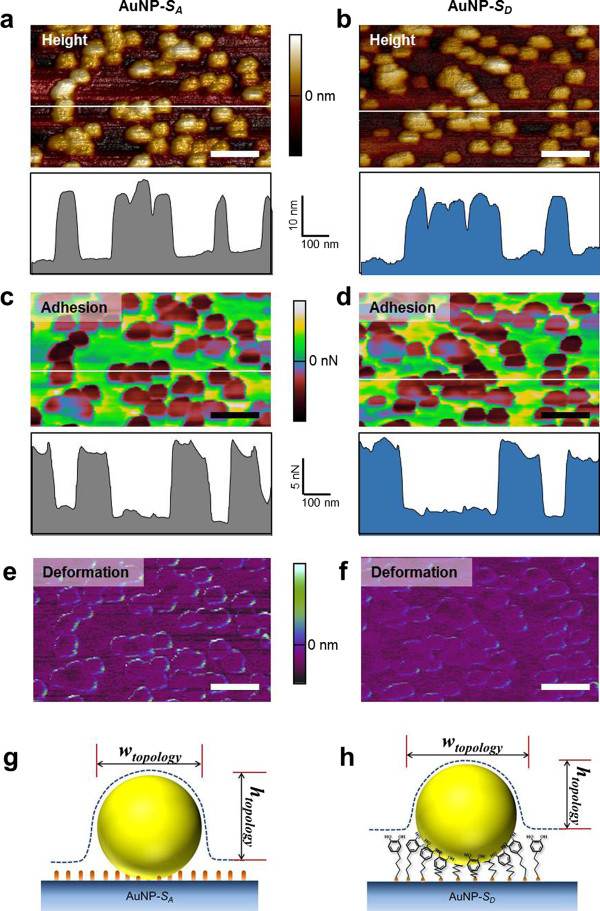
**PeakForce QNM analysis of AuNP-immobilized surfaces.** (**a**, **b**) Topographic AFM images of 20 nm of AuNPs immobilized on aminated surface and DOPA-functionalized surface, respectively. (**c, d**) Adhesion images of the samples show relative adhesion interaction between the bare Si AFM tip and AuNPs or other regions outside the AuNPs. All scale bars are 200 nm. The dashed line is a depiction of AFM stylus trajectory. (**e, f**) AFM deformation images show a larger deformation change near the edge than at the center of the nanoparticles. (**g, h**) Schematic diagram of the geometric design depicted AuNPs immobilized on the substrates functionalized with amino group and DOPA molecules, respectively. The terms *w*_topology_ and *h*_topology_ indicate the measured width and height, respectively, of the AuNPs in the aspect of topology (a, b).

While measurement of the number of attached AuNPs on the surface or mass of the AuNPs bound to the surface is an indirect method to quantify the binding affinity between AuNPs and chemically functionalized surface, we have taken into account the direct method for quantitative characterization of such binding affinity. Here, we have employed a novel scanning technique, namely PeakForce QNM [49], that is useful in measuring the adhesion force between AuNPs and chemically modified surface. Figure 4a,b shows the AFM topography images of AuNPs attached to either *S*_A_ or *S*_D_. It is shown that the AFM height for the AuNPs attached to *S*_D_ is measured as approximately 14.2 nm, whereas the AFM height for the AuNPs bound to *S*_A_ is measured as approximately 15.5 nm. This is attributed to the size of the functionalized molecules such that the chain length of DOPA is much larger than that of the amine group [48]. As shown in Figure 4g,h, the AuNP is more likely to be embedded in DOPA, that is, more number of DOPA molecules (than that of amine molecules) is likely to be involved in AuNP binding. This suggests that DOPA molecules may allow for establishing the stable, reliable adhesion of nanoparticles. Notably, we found that the width of the AuNPs bound to *S*_D_ (99.1 ± 12.3) in both topology and adhesion map is larger by the amount of approximately 7 nm than that bound to *S*_A_ (92.2 ± 19.7), as shown in Figure 4a,b. This result seems to contradict the fact that the AuNPs in *S*_D_ are more deeply embedded than those in *S*_A_. It may be attributed to the fact that AFM indentation may induce the significant motion of AuNPs, which may distort the size of the AuNPs. In particular, a previous study [50] reports that there is greater energy dissipation at the edge of a nanoparticle than at its center, implying that the nanoparticle would be wobbled during AFM indentation, whereas the nanoparticle would not be moved during tapping mode AFM imaging. As shown in the AFM deformation images (Figure 4e,f), there is a larger deformation change of AuNPs in AuNP-*S*_A_ than in AuNP-*S*_D_ during PeakForce QNM. This indicates a stronger binding of AuNPs onto *S*_D_ rather than on *S*_A_, which is attributed to the narrow structural dimension of AuNPs immobilized on *S*_A_ in comparison with those on *S*_D_. Moreover, we have also considered the adhesion map for AuNPs attached to *S*_A_ or *S*_D_. It is found that the adhesion force difference between silicon nitride (Si_3_N_4_) AFM tip and the surface (i.e., *S*_A_ or *S*_D_) is <5 nN and that the adhesion force is not significantly dependent on the type of surface chemistry (i.e., whether the surface is functionalized with amine group or DOPA). This indicates that the interaction between the Si_3_N_4_ AFM tip and the surface is not critical when we measure the adhesion force between AuNPs and surface. It is shown that the adhesion force between the Si_3_N_4_ AFM tip and chemically modified surface to which the AuNPs do not adhere is measured as approximately 10 nN. Nevertheless, the adhesion force map obtained from PeakForce QNM is insufficient to distinguish the binding affinity between AuNPs and *S*_D_ from that between AuNPs and *S*_A_, while the AFM height in topology and the width in the adhesion map for AuNPs bound to *S*_A_ or *S*_D_ allow for the distinction between such binding affinities as described earlier. 

**Figure 5:**
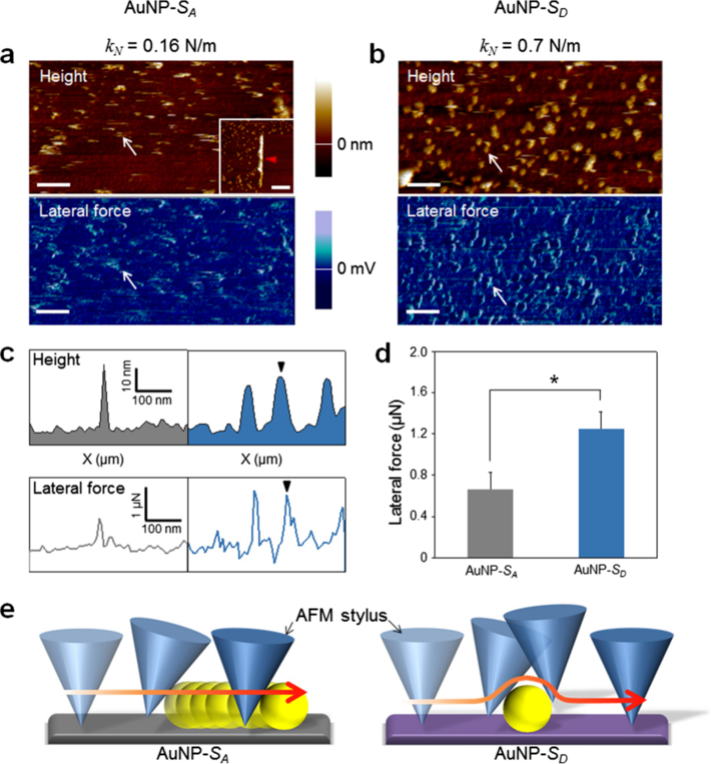
**Lateral force microscopy analysis of binding affinity between AuNPs and chemically functionalized surfaces. **(**a**, **b**) AFM topographic images and lateral force images of AuNPs immobilized on Si substrates, respectively, functionalized with (a) amino groups (AuNP-*S*_A_) and (b) catecholamine molecules (AuNP-*S*_D_) by different normal spring constants (*k*_N_) of microcantilevers. The inset shows left embankment (indicated by a red arrowhead) due to swept AuNPs formed by scanning the surface in AFM contact mode with *k*_N_ = 0.7 N/m of microcantilever. All scale bars including that of the inset are 250 nm. (**c**) Line profiles corresponding to the white arrow in each image of (a) and (b) show the curves of scanner retracting distance versus the AuNP lateral displacement and the lateral force versus the lateral displacement, respectively. (**d**) Graph of the average lateral force of >100 AuNPs in (a) and (b) (the asterisk indicates *p* < 0.001) was extracted and calculated from the line profiles of lateral forces (c). (**e**) The model illustrates the physical interaction between the AFM tip and AuNPs attached on the chemically functionalized substrate (i.e., AuNP-*S*_A_ and AuNP-*S*_D_).

Another way to directly measure the binding affinity between AuNPs and chemically functionalized surface is to utilize LFM that enables the measurement of friction force between the AFM tip and the sample surface (Figure 5). For LFM imaging, we have utilized a triangular-shaped microcantilever whose normal spring constant *k*_norm_ is in the range of 0.16 to 1 N/m, suitable for contact mode AFM imaging. In general, the normal spring constant of a cantilever depends on its shape and material [51]. In our study, we have used Si_3_N_4_ cantilevers so that the normal spring constant of a microcantilever is determined from its shape. The lateral spring constant *k*_lat_ is related to the normal spring constant given by the following equation [35]:

(1) $$k_{\mathrm{lat}}=\frac{2}{6\cos^{2}\theta +3\left(1+v\right)\sin^{2}\theta }{\left(\frac{L}{H}\right)}^{2}k_{\mathrm{norm}}$$

where *θ* is the angle between the base arms of the triangular cantilever, *v* is the Poisson ratio for silicon nitride, *L* is the length of the cantilever beam, and *H* is the tip vertical height (see Table 2). With the estimation of *k*_lat_ from Equation 1, the lateral force (*F*_lat_) can be calculated from the measurement of the lateral force signal in LFM analysis such as [36]

(2) $$F_{\mathrm{lat}}=k_{\mathrm{lat}}\times S_{\mathrm{lat}}\times \Delta V$$

**Table 2:** Summary of triangular microcantilever parameters (SNL and ScanAsyst Fluid) used in LFM study

	**ScanAsyst Fluid**	**SNL (B)**
*L* (μm)	70	205
*w* (μm)	10	40
*t* (μm)	0.6	0.6
*H* (μm)	8	8
*E* (GPa)	304	304
*ν*	0.24	0.24
*θ*	60°	64.5°
*k*_norm_ (N/m)	0.74	0.16
*S*_norm_ (nm/V)	12.57	26.30
*k*_lat_ (N/m)	19.57	56.16
*S*_lat_ (nm/V)	1.88	1.27

*L*, cantilever length; *w*, cantilever width; *t*, cantilever thickness; *H*, stylus height; *E*, Young's modulus of Si; *v*, Poisson's ratio of Si; *θ*, angle between the base arms of the triangular cantilever; *k*_norm_, measured cantilever's normal spring constant; *S*_norm_, measured cantilever's normal sensitivity; *k*_lat_, lateral spring constant calculated from the *k*_norm_; *S*_lat_ lateral normal sensitivity calculated from *S*_norm_[35,36].

Here, *S*_lat_ is the lateral sensitivity of the cantilever defined as *S*_lat_ = *PHS*_norm_/aR*L[36], where *P* is a proportionality factor (≈2.5 for the triangular cantilever), *S*_norm_ is the vertical deflection sensitivity of the cantilever, *a* is the amplification factor of the lateral signal measured, and R* is the ratio of the beam height to the beam width (R* = 0.5) [36]. Δ*V* is the measured value in LFM analysis, which is extracted from the LFM images. In general, the longer and larger the cantilever, the lower is its normal spring constant (i.e., more flexible in normal deflection), but the larger is its lateral spring constant (*k*_lat_). We can control the exerted applied force using different spring constants of cantilevers (*k*_norm_) under an identical deflection set point (1 V) rather than a set point control with an identical AFM tip in order to avoid damage to the samples and a subsidiary frictional noise.

**Figure 6:**
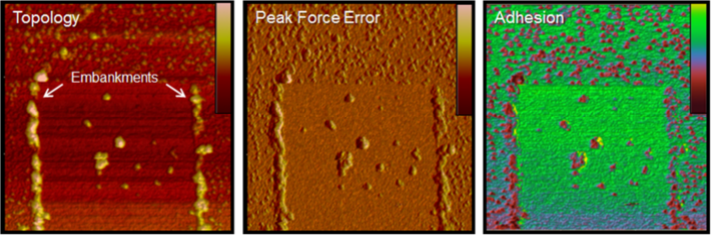
**The PeakForce QNM analysis of the AuNP-*S***_**D**_**sample.** AFM topology, peak force error, and adhesion images (3 × 3 μm^2^) of the embankments composed of AuNPs swept by the 2 × 2 μm^2^ scanning of a microcantilever with *k*_N_ = 1 N/m in LFM.

**Figure 7:**
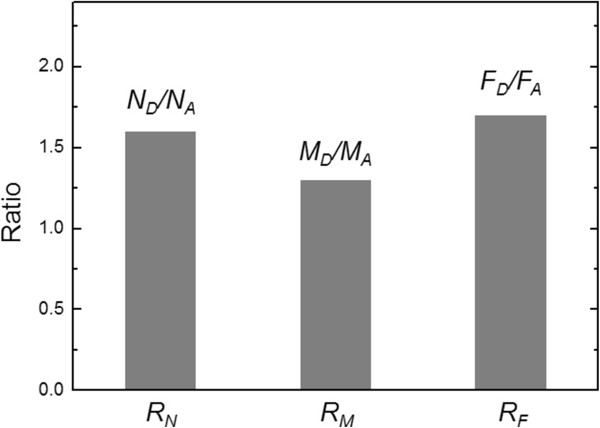
**Plot of binding affinity ratio between AuNPs and chemically functionalized surfaces (AuNP-*S***_**A**_**and AuNP-*S***_**D**_**).** The binding affinity ratios are obtained from experiments such as SEM image, cantilever assay, and LFM. This analysis indicates that DOPA molecules are approximately 1.5 times stronger than amino groups in their adhesion property for the immobilization of AuNPs.

In LFM imaging for the measurement of friction force between the two surfaces (i.e., surface of the AuNPs and the substrate functionalized with chemical groups), one has to be cautious in selecting a cantilever; in particular, a cantilever with a stiffness of <0.16 N/m is too flexible to scan our sample, whereas a cantilever with a stiffness of ≥1 N/m is too stiff to measure the friction force in our sample. As shown in Figure 6, the AFM imaging of our sample using a cantilever with a stiffness of 1 N/m leads to the detachment of AuNPs from the surface during the imaging, which implies the difficulty in accurately measuring the friction force between AuNPs and surface (i.e., *S*_A_ or *S*_D_). Figure 5a,b shows the AFM/LFM images of AuNPs attached to *S*_D_ or *S*_A_. It is shown in Figure 5a that during AFM imaging using a cantilever with a stiffness of *k*_norm_ = 0.16 N/m, AuNPs are detached from *S*_A_ (i.e., AFM image shows a scratched pattern corresponding to the imaged AuNPs), while the detachment of AuNPs from *S*_D_ does not occur. Moreover, it is found that AuNPs are still bound to *S*_D_ even when AFM and LFM imaging were implemented using a cantilever with a stiffness of *k*_norm_ = 0.7 N/m (Figure 5b). Figure 5c shows the section profile extracted from the AFM/LFM images (as indicated by a white arrow). It is shown that in the AFM height profile, as anticipated, the AFM height of the AuNPs bound to *S*_A_ is close to that of the AuNPs attached to *S*_D_. On the other hand, in the lateral force profile extracted from the LFM image, we can find the significant differences between the durability of two samples, i.e., AuNPs bound to *S*_A_ and *S*_D_, respectively. This is attributed to the fact that during imaging of AuNPs bound to *S*_A_, the twist of the cantilever tip is not significant, which leads to low signals in the LFM image, while the binding between AuNPs and *S*_D_ (stronger than that between AuNPs and *S*_*A*_) leads to more twist of the cantilever tip and consequently produce a large signal in LFM imaging [37] (Figure 5e). Based on the LFM images with Equations 1 and 2, we have measured the lateral force between AuNPs and chemically modified surface. It is found that the mean lateral force between AuNPs and *S*_A_ is measured as 660 nN, while the mean lateral force between AuNPs and *S*_D_ is estimated to be 1.2 μN (Figure 5d). For quantitative comparison, we have introduced a dimensionless parameter defined as *R*_F_ = *F*_D_/*F*_A_, where *F*_A_ indicates the lateral force between AuNPs and *S*_A_, and *F*_D_ represents the lateral force between AuNPs and *S*_D_. It is interestingly found that the dimensionless parameter *R*_F_ (=1.7) is very close to the aforementioned dimensionless parameters *R*_N_ and *R*_M_ (Figure 7). This suggests that the binding affinity between AuNPs and chemically functionalized surface can be quantitatively understood by using either of the indirect experimental methods such as cantilever assay or direct force measurement such as LFM imaging. It should be noted that the binding force between AuNPs and chemically functionalized surface could be measured using AFM pulling experiments [35,52], which enables the measurement of the normal force required to break a chemical bond. In general, the normal adhesion force driven by the mechanical detachment of AuNPs from the surface might be much lower than the shear adhesion force between AuNPs and the surface. It is attributed to the fact that a shear force required to break chemical bonds is much larger than a normal force that leads to breakage of chemical bonds [53,54]. This indicates that LFM imaging-based measurement allows for estimating the maximum strength of chemical bonds between nanostructure and chemical functional group. Moreover, AFM pulling experiment-based measurement of normal force required to break chemical bonds requires statistical analysis (based on repetitive experiments due to the effect of thermal fluctuation on force-driven bond rupture [55-57]), while LFM imaging-based measurement of shear force for breaking bonds does not require repetitive experiments because LFM imaging enables the parallel measurement of shear forces required to break chemical bonds in the scanned area of a sample. In other words, LFM imaging enables the simultaneous measurement of shear forces (with more than 100 times) required to break chemical bonds, which results in an effective statistical analysis based on only a single LFM image. 

## Conclusions

In conclusion, we have demonstrated a quantitative characterization of the binding affinity between AuNPs and chemically modified surface using various experimental techniques such as SEM image analysis, cantilever assay, PeakForce QNM, and LFM image analysis. It is shown that the DOPA-modified surface is an effective conjugation method for functionalization of nanoparticles onto the surface when compared with amine-modified surface, as anticipated, from our various experiments. More remarkably, we have shown that dimensionless parameters (i.e., *R*_N_, *R*_M_, and *R*_F_) introduced in this work are useful in quantifying the binding affinity between nanoparticle and chemical functional groups, and that these dimensionless parameters are consistent regardless of experiments, i.e., *R*_N_, *R*_M_, and *R*_F_ are almost identical to each other, implying that the binding affinity between nanostructure and chemical group can be quantitatively studied using either indirect method (i.e., SEM image analysis and cantilever assay) or direct method (i.e., lateral force measurement). Our study sheds light on how to quantitatively study the binding affinity between nanostructure and chemical functional group, which can provide the design principles for nanoparticle-based systems such as nanomedicine and nanobiosensor.

## Abbreviations

AFM: Atomic force microscopy; APTMS: 3-aminopropyltrimethoxysilane; AuNP: Gold nanoparticle; DOPA: Dopamine hydrochloride; PeakForce QNM: PeakForce quantitative nanomechanical property mapping; LFM: Lateral force microscopy; *S*_A_: Amine-functionalized surface; *S*_D_: DOPA-modified surface; SEM: Scanning electron microscope; XPS: X-ray photoelectron spectroscopy.

## Competing interests

The authors declare that they have no competing interests.

## Authors’ contributions

GL carried out the experiments and wrote the manuscript. HL performed the experiments. KN and JHH participated in the XPS and SEM analyses and in the interpretation of data. JY, SWL, and DSY participated in the discussion and interpretation of data. KE participated in the analysis of the experimental data, interpreted the result, and wrote the manuscript. TK conceived the research, analyzed the experimental data, interpreted the results, and wrote the manuscript. All authors read and approved the final manuscript.
